# Cyclic AMP stimulates platelet-derived growth factor B chain mRNA expression in murine macrophage cell lines

**DOI:** 10.1155/S0962935195000470

**Published:** 1995-07

**Authors:** Elizabeth J. Kovacs, Susan VanStedum

**Affiliations:** 1 Department of Cell Biology, Neurobiology, and Anatomy Loyola University Maywood IL 60153 USA; 2 the Burn and Shock Trauma Institute Loyola University Maywood IL 60153 USA; 3 the Molecular Biology Program Loyola University Maywood IL 60153 USA

## Abstract

Prostaglandin E_2_ plays a role in cytokine production presumably by altering intracellular levels of cAMP. In this paper, we report on the differential expression of cytokine genes in murine macrophages in response to stimulation with activators of cAMP. Macrophages were cultured with or without cAMP activators in the presence or absence of LPS. Prior to treatment, macrophages do not express interleukin-1β, but do express low levels of tumour necrosis factor α and platelet-derived growth factor B chain mRNAs. After culture with cAMP-inducers, including PGE_2_, dibutyryl cAMP and forskolin, PDGF B chain mRNA is induced. Forskolin, for example, induced expression PDGF B chain mRNA to a level ranging from 25% to 200% of the level induced by LPS in 6 h. In contrast, cAMP-inducers enhance the expression of IL-1β and TNF-α mRNAs, but only in the presence of LPS. The combination of forskolin and LPS does not appear to act synergistically on PDGF B chain mRNA levels, suggesting that LPS-stimulated effects are not mediated through a cAMP-dependent pathway. Furthermore, macrophages differentially express cytokine genes in response to treatment with inducers of intracellular cAMP.


Mediators of Inflammation 4, 293–297 (1995)

### Cyclic AMP stimulates platelet-derived growth factor B chain mRNA expression in murine macrophage cell lines

Elizabeth J. Kovacs[1,2,3,CA] and Susan VanStedum[1]

[1]Department of Cell Biology, Neurobiology, and Anatomy; the [2]Burn and Shock Trauma Institute; and the [3]Molecular Biology Program, Loyola University, Maywood, IL 60153, USA

[CA]Corresponding Author

**PROSTAGLANDIN $E_2$ plays a role in cytokine production presumably by altering intracellular levels of cAMP. In this paper, we report on the differential expression of cytokine genes in murine macrophages in response to stimulation with activators of cAMP. Macrophages were cultured with or without cAMP activators in the presence or absence of LPS. Prior to treatment, macrophages do not express interleukin-1β, but do express low levels of tumour necrosis factor α and platelet-derived growth factor B chain mRNAs. After culture with cAMP-inducers, including $PGE_2$, dibutyryl cAMP and forskolin, PDGF B chain mRNA is induced. Forskolin, for example, induced expression PDGF B chain mRNA to a level ranging from 25% to 200% of the level induced by LPS in 6 h. In contrast, cAMP-inducers enhance the expression of IL-1β and TNF-α mRNAs, but only in the presence of LPS. The combination of forskolin and LPS does not appear to act synergistically on PDGF B chain mRNA levels, suggesting that LPS-stimulated effects are not mediated through a cAMP-dependent pathway. Furthermore, macrophages differentially express cytokine genes in response to treatment with inducers of intracellular cAMP.**

**Key words:** cAMP, Gene expression, Lipopolysaccharide, Macrophages, Platelet-derived growth factor.

### Introduction

A variety of diseases characterized by fibrosis share common elements. The pathogenesis of these disorders includes the proliferation of fibroblasts and the deposition of extracellular matrix (for a review see Reference 1). This often begins as an inflammatory reaction with leukocyte infiltration followed by the elaboration of cytokines. In the absence of inhibitory signals, the aberrant production of these mediators sustains the connective tissue accumulation which results in permanent alteration in tissue structure and function.

Macrophages play a central role in the tissue response to injury by releasing cytokines and mediate immune-mesenchymal cell interactions and trigger the proliferation fibroblasts and the deposition of connective tissue.[2] Although the mechanisms responsible for the activation of macrophages are not fully understood, evidence suggests that soluble factors including cytokines and arachidonic acid metabolites, including prostaglandin $E_2$ ($PGE_2$), play a critical role in the process.[3,4] We have previously reported on the induction of expression of PDGF genes in rat peritoneal macrophage in response to treatment with cytokines, including interleukin-2,[5] and herein examine the role of $PGE_2$ and agents which stimulate the accumulation of intracellular cAMP on the expression of the PDGF B chain gene in the murine macrophage cell line ANA-1.

### Materials and Methods

*Reagents:* Forskolin, dibutyryl-cAMP (dBcAMP), 8-bromo-cAMP, isomethyl-3-isobutylxanthine (IBMX), cholera toxin, prostaglandin $E_2$ ($PGE_2$) and dibutyryl-cGMP (dBcGMP) were obtained from Sigma Chemicals (St. Louis, MO, USA). Lipopolysaccharide (LPS) was purchased from Difco (Detroit, MI, USA).

*Culture of cells:* The ANA-1 murine macrophage cell line was obtained from Dr Luigi Varesio (NCI-FCRDC, Frederick, MD, USA).[6] Prior to stimulation, cells were washed and incubated at $1 \times 10^6$ cells/ml in RPMI 1640 containing 1% FBS (with penicillin sulfate, streptomycin and glutamine) with or without agents which alter intracellular cAMP in the presence or absence of LPS for various periods of time.

© 1995 Rapid Science Publishers

*RNA isolation and hybridization:* Total cellular RNA was isolated using the RNAzol Method (Biotecx, Houston, TX, USA) according to the manufacturer's specifications. Northern blot analysis was performed with 10 µg of RNA and probed with cDNA probes labelled by random priming with [$^{32}$P]-dCTP as described previously.[7] Cells were maintained as a semi-adherent culture in RPMI 1640 medium supplemented with 10% fetal bovine serum (FBS), 100 µ/ml penicillin sulfate, 100 µg/ml streptomycin, and 2 mM glutamine (GIBCO, Grand Island, NY, USA) and passages. cDNA probes used in these studies were obtained from the following sources: PDGF B chain (c-sis) from ATCC (Rockville, MD, USA); human TNF-α from Dr S. Socher (Merck, Sharp & Dohme Res. Labs., West Point, PA, USA); human IL-1β from Dr D. Carter (The Upjohn Co, Kalamazoo, MI, USA); and chicken β-actin cDNA from Dr D.W. Cleveland (Johns Hopkins Univ., Baltimore, MD, USA). Equivalent amounts of total RNA/lane were assessed by monitoring 28S and 18S ribosomal RNA.

*Immunoblot analysis of cytokine protein levels:* To determine cytokine protein levels, immunoblot analysis was performed as previously described[8] with the following minor modifications. Supernatants (100 µl) from cultured cells were blotted on nitrocellulose filters. The filters were blocked with gelatin and sequentially incubated with anti-human PDGF BB antibody (Genzyme, Cambridge, MA) followed by biotin-conjugated anti-rabbit IgG. The filters were then incubated with avidin-conjugated alkaline phosphatase followed by a substrate for colour development.

Data presented herein are representative examples of experiments performed a minimum of three times unless otherwise specified.

### Results

*$PGE_2$ and cAMP inducers elevation of PDGF B chain mRNA expression:* Treatment of ANA-1 macrophages with $PGE_2$ stimulates the expression of PDGF B chain mRNA (Fig. 1). PDGF B chain mRNA is expressed in ANA-1 cells following culture with as little as 2 ng/ml of $PGE_2$ and peak levels were induced at 20 ng/ml of $PGE_2$. Similar results were obtained after incubation of ANA-1 cells with forskolin, which is known to stimulate adenylate cyclase activity.[9] In contrast, LPS (1 µg/ml) minimally induces expression of PDGF B chain mRNA.

*Differential expression of cytokines genes in response to treatment with LPS and forskolin:* To determine whether the induction of cytokine mRNA expression was specific for PDGF B chain, we monitored the expression of IL-1β and TNF-α in ANA-1 cells cultured for 4 h with either LPS alone, forskolin alone or both LPS and forskolin (Fig. 2). Northern blots revealed that only PDGF B chain mRNA expression was stimulated by forskolin alone. In contrast to PDGF B chain mRNA, the expression of IL-1β and TNF-α mRNAs were greatly induced by stimulation with LPS alone. Furthermore, treatment of ANA-1 cells with LPS and forskolin only yielded slightly higher levels of IL-1β and TNF-α mRNAs than LPS alone, further suggesting a stimulatory role for a cAMP-dependent pathway in the control of PDGF B chain gene expression.

FIG. 1. Effects of $PGE_2$ and agents which elevate intracellular cAMP on the expression of PDGF B chain mRNA. ANA-1 cells were cultured with or without inducing for 6 h. Blots were hybridized sequentially with probes for PDGF B chain and actin.

A quantitative analysis of the expression of PDGF B chain and TNF-α mRNAs is shown in Fig. 3. Blots were prepared from ANA-1 cells treated with either LPS alone, forskolin alone or both LPS and forskolin for 4 h. Scanning densitometric analysis (Fig. 3) revealed that PDGF B chain mRNA was induced over 40-fold following treatment with forskolin, and that this level was not further enhanced by LPS. The induction of TNF-α mRNA expression by LPS was over 19-fold and was enhanced to 17-fold by the addition of forskolin to LPS. LPS alone marginally induced PDGF B chain mRNA expression, as did forskolin alone for TNF-α mRNA levels.

*Time course of induction of expression PDGF B chain mRNA:* ANA-1 cells were cultured with forskolin (10 µM) for various periods of time before harvest of cells for RNA isolation and blot

FIG. 2. Differential expression of cytokine genes in response to treatment with LPS and forskolin. ANA-1 cells were cultured for 4 h with or without LPS (1 μg/ml) and forskolin (10 μM). Northern blots were sequentially hybridized with the indicated cDNA probes.

FIG. 3. Expression of PDGF B chain and TNF-α mRNAs. Blots were prepared from ANA-1 cells following 4 h of culture with or without LPS (1 μg/ml) in the presence or absence of forskolin (10 μM). Blots containing total cellular RNA were hybridized probes for PDGF B chain, TNF-α and actin. Quantification of mRNA expression was made by scanning densitometric analysis. Data are reported as arbitrary optical (O.D.) units.

analysis. PDGF B chain mRNA is marginally expressed in untreated cells and is rapidly induced within 1 h of treatment with forskolin. Maximal expression is achieved between 4 and 18 h of culture, after which the PDGF B chain mRNA level gradually declines.

*Modulation of PDGF B chain mRNA expression by agents which elevate intracellular cAMP levels:* To confirm that the stimulatory effects of forskolin on the expression of PDGF B chain mRNA expression were due to the enhancement of cAMP levels, ANA-1 cells were cultured in the presence or absence of several agents which elevate intracellular cAMP. These include $PGE_2$ forskolin, dBcAMP, 8-bromo-cAMP, IBMX (an inhibitor phosphodiesterase and, thus, indirectly enhancing intracellular levels of cAMP by blocking its degradation),[10] and cholera toxin (which triggers continuous activation of adenylate cyclase by altering the α subunit of the stimulatory GTP-binding protein).[11] All of the agents which triggered the elevation of intracellular cAMP stimulated the enhanced expression of PDGF B chain mRNA (Fig. 4). In contrast, the addition of dBcGMP to ANA-1 cells failed to stimulate levels of PDGF B chain mRNA above background (not shown).

*Effects of cAMP-inducing agents on PDGF B chain protein production:* Immunoblot analysis was performed to determine whether incubation of ANA-1 with cAMP-inducing agents triggered the production and secretion of PDGF B chain protein. Fig. 5 shows that PDGF B chain protein is released by macrophages following culture with a variety of agents which trigger the accumulation of intracellular cAMP. In contrast, treatment with a cGMP-inducer fails to induce ANA-1 cells to secrete detectable levels of PDGF B chain protein.

### Discussion

The expression of PDGF-like molecules by macrophages in response to tissue injury has been well documented (for a review, see Refer-

FIG. 4. Comparison of effects of agents which elevate cAMP on PDGF B chain mRNA expression and protein production. ANA-1 cells were cultured for 4 h with or without the following agents: forskolin (10 μM), LPS (1 μg/ml), dBcAMP (0.1 μM), 8-bromo-cAMP (0.1 μM), IBMX (0.2 mM), $PGE_2$ (20 ng/ml), and cholera toxin (5 μM). Northern blot analysis reveals that a variety of agents which induce the accumulation of cAMP trigger the expression of PDGF B chain mRNA.

FIG. 5. Secretion of PDGF B chain protein by ANA-1 cells. Immunoblot analysis of PDGF B chain protein indicates that PDGF B chain protein is produced and secreted by macrophages following treatment with cAMP-inducing agents.

ence 12). Alveolar macrophages from normal lungs do not spontaneously release PDGF-like mediators, but can be induced to secrete these cytokines following *in vitro* treatment with a variety of agents.[13–16] In contrast, constitutive production of high levels of PDGF is observed in macrophages isolated from the lungs of animals undergoing the repair process following pulmonary injury induced by exposure to cytotoxic drugs,[17] asbestos[18] and hyperoxia,[19] as well as patients with idiopathic pulmonary fibrosis,[20–22] adult respiratory disease syndrome,[23] and scleroderma lung disease (EJ Kovacs *et al.*, unpublished observation). In addition, alveolar lining fluid collected by pulmonary lavage from bleomycin treated rats[24] and patients with pulmonary fibrosis,[25] contains PDGF-like cytokines, suggesting their release *in situ*.

The involvement of macrophage-derived fibrogenic cytokines, such as PDGF, in the development of fibrosis is not restricted to the lung. PDGF has been found in the peritoneal fluid of patients with endometriosis[26] as well as in patients undergoing peritoneal dialysis[27] or intraperitoneal immunotherapy for the treatment of abdominal tumours.[28] In addition, hyperplasia of smooth muscle cell and accumulation of extracellular matrix along with a lipid component are associated with the development of atherosclerosis.[29] This is believed to result from the release of a PDGF-like mediator from infiltrating macrophages.[30] Finally, human wound fluid contains PDGF-like molecules.[31] At present, it is not clear whether the immunoreactive PDGF described herein is identical to the platelet-derived molecules or similar to the smaller molecular weight peptides recently described.[23,31]

While these disorders all share the common denominator of local PDGF release by macrophages, it is unclear what triggers the local production. Since the release of prostaglandins, including $PGE_2$, occurs during inflammatory/prefibrotic responses, we hypothesized that $PGE_2$ could act in an autocrine/paracrine feed-forward manner to trigger the production of PDGF B chain by macrophages.

Our data are in concurrence with other studies demonstrating a stimulatory role for $PGE_2$ and cAMP-inducers in the control of a subset of macrophage functions, including cholesterol ester clearance[32,33] and IL-6 production.[34] The role of cAMP in the regulation of IL-1 and TNF-α genes has remained somewhat controversial, with some laboratories reporting that cAMP inducers inhibit IL-1 and TNF-α gene expression, while others state that they stimulate expression (for a review, see Reference 35).

With regard to the transcription of IL-1 genes, studies performed in murine peritoneal macrophages demonstrate an inhibitory role for cAMP and cAMP-inducers.[36,37] However, in human monocytes, it acts primarily as a stimulant.[38–40] Our data reveal that the cAMP inducer, forskolin, has a marginal stimulatory effect on LPS-induced expression of IL-1 mRNA in the fetal liver-derived ANA-1 murine macrophage cell line (Fig. 2). Thus, the action of cAMP-inducing agents on the production of IL-1 seems to be species, as well as cell type, specific.

In conclusion, these data demonstrate that agents which trigger the accumulation of intracellular cAMP specifically stimulate the expression of the PDGF B chain gene, without altering the endogenous or LPS-induced expression of TNF-α or IL-1β mRNA. Hence, the expression of cytokine genes may be divided into two classes, ones (such as PDGF B chain) which are activated by inducers of cAMP and others (including TNF-α and IL-1β) which are not.

### References

1. Kovacs EJ. Fibrogenic cytokines: The role of TGFβ and PDGF in the development of fibrosis. *Immunol Today* 1991; **12:** 17–23.
2. Leibovich SJ, Ross R. The role of macrophages in wound repair: A study with hydrocortisone and anti-macrophage serum. *Am J Physiol* 1975; **78:** 71–100.
3. Bonney RJ, Burger S, Davies P, Kuehl FA Jr, Humesand JL. Prostaglandin $E_2$ and prostacyclin elevate cyclic AMP levels in elicited populations of mouse peritoneal macrophages. *Adv Prostaglandin Thromboxane Res* 1980; **8:** 1691–1693.
4. Yamamoto H, Suzuki T. Prostaglandin $E_2$-induced activation of adenosine 3′-5′ cyclic monophosphate-dependent protein kinases of a murine macrophage-like cell line ($P388D_1$). *J Immunol* 1987; **139:** 3416–3421.
5. Kovacs EJ, Neuman JE. Selective induction of PDGF A chain and B chain gene expression in rat peritoneal macrophages by interleukin-2. In: Meltzer MM, Mantovani, A, eds. *Cellular and Cytokine Networks in Tissue Immunity.* New York: John Wiley & Sons. 1991; 307–312.
6. Cox GW, Matheison BL, Gandino L, Blasi E, Varesio L. Heterogeneity of hematopoietic cells immortalized by v-myc/v-raf recombinant retrovirus infection of bone marrow or fetal liver. *J Natl Cancer Inst* 1988; **81:** 1492–1495.
7. Kovacs EJ, Oppenheim JJ, Young HA. Induction of c-fos and c-myc expression in T lymphocytes following treatment with IL-1α. *J Immunol* 1986; **137:** 3649–3651.
8. Walsh MJ, LeLeiko NS, Sterling KM Jr. Regulation of types I, III, and IV procollagen mRNA synthesis in glucocorticoid-mediated intestinal development. *J Biol Chem* 1987; **262:** 10814–10818.
9. Seamon KB, Padgett W, Daly JW. Forskolin: unique diterpene activator of adenylate cyclase in membranes and intact cells. *Proc Natl Acad Sci* 1981; **78:** 3363–3367.
10. Wells JN, Kramer GL. Phosphodiesterase inhibitors as tools in cyclic nucleotide research. *Molec Cell Endocrinol* 1981; **23:** 1–9.
11. Cassel D, Pfeuffer T. Mechanism of cholera toxin action: covalent modification of the guanine nucleotide-binding protein of the adenylate cyclase system. *Proc Natl Acad Sci* 1978; **75:** 2669–2673.
12. Kelley J. Cytokines of the lung. *Amer Rev Respir Dis* 1990; **141:** 765–788.
13. Martinet Y, Bitterman PB, Mornex J-F, Grotendorst GR, Martin GR, Crystal RG. Activated human monocytes express the c-sis proto-oncogene and release a mediator showing PDGF-like activity. *Nature* 1986; **319:** 158–160.
14. Bauman MD, Jetten AM, Brody AR. Biologic and biochemical characterization of a macrophage-derived growth factor for rat lung fibroblasts. *Chest* 1987; **91:** 15S–16S.
15. Kumar RK, Bennett RA, Brody AR. A homologue of platelet-derived growth factor produced by rat alveolar macrophages. *FASEB J* 1988; **2:** 2272–2277.
16. Antoniades HN, Bravo MA, Avila RE, *et al.* Platelet-derived growth factor in idiopathic pulmonary fibrosis. *J Clin Invest* 1990; **86:** 1055–1064.
17. Kovacs EJ, Kelley J. Lymphokine regulation of macrophage-derived growth factor secretion following pulmonary injury. *Am J Pathol* 1985; **121:** 261–268.
18. Bauman MD, Jetten AM, Bonner JC, Kumar RK, Bennett RA, Brody AR. Secretion of a platelet-derived growth factor homologue by rat alveolar macrophages exposed to particulates *in vitro. Eur J Cell Biol* 1990; **51:** 327–334.
19. Fabisiak JP, Evans JN, Kelley J. Increased expression of PDGF-B (c-sis) mRNA in rat lung precedes DNA synthesis and tissue repair during chronic hyperoxia. *Am J Respir Cell Molec Biol* 1989; **1:** 181–189.
20. Mornex JF, Martinet Y, Yamauchi K, *et al.* Spontaneous expression of the c-sis gene and release of a platelet-derived growth factor like molecule by human alveolar macrophages. *J Clin Invest* 1986; **78:** 61–66.
21. Martinet Y, Rom WN, Grotendorst GR, Martin GR, Crystal RG. Exaggerated spontaneous release of platelet-derived growth factor by alveolar macrophages from patients with idiopathic pulmonary fibrosis. *N Engl J Med* 1987; **317:** 202–209.
22. Nagaoka I, Trapnell BC, Crystal RG. Upregulation of platelet-derived growth factor A and B chain gene expression in alveolar macrophages of individuals with idiopathic pulmonary fibrosis. *J Clin Invest* 1990; **85:** 2023–2027.
23. Snyder LS, Hertz MI, Peterson MS, *et al.* Acute lung injury: pathogenesis of intra-alveolar fibrosis. *J Clin Invest* 1991; **88:** 633–673.
24. Kovacs EJ, Kelley J. Lavage assesses the mitogenic state of the lung: intra-alveolar release of growth factor following lung injury. *Amer Rev Respir Dis* 1986; **133:** 68–72.
25. Yamauchi K, Martinet Y, Basset P, Fells GA, Crystal RG. High levels of transforming growth factor-β are present in the epithelial lining fluid of the normal lower respiratory tract. *Amer Rev Res Dis* 1988; **137:** 1360–1363.
26. Halme J, White C, Kauma S, Estes J, Haskill S. Peritoneal macrophages from patients with endometriosis release growth factor activity *in vitro. J Clin Endo Metab* 1988; **66:** 1044–1049.
27. Shimokado K, Raines EW, Madtes DK, Barrett TB, Benditt EP, Ross R. A significant part of macrophage-derived growth factor consists of at least two forms of PDGF. *Cell* 1985; **43:** 277–286.
28. Kovacs EJ, Brock B, Silber IE, Neuman JE. LAK cell expression of PDGF and TGF-β mRNAs. In: Podwanda MC, Oppenheim JJ, Kluger M, Dinarello CA, eds. *Molecular and Cellular Biology of Cytokines.* New York: Alan R Liss. 1990; 407–412.
29. Ross R. The pathogenesis of atherosclerosis—an update. *N Eng J Med* 1986; **314:** 488–500.
30. Ross R, Masuda J, Raines EW, *et al.* Localization of PDGF-B protein in macrophages in all phases of atherosclerosis. *Science* 1990; **248:** 1009–1012.
31. Matsuoka J, Grotendorst GR. Two peptides related to platelet-derived growth factor are present in human wound fluid. *Proc Natl Acad Sci* 1989; **86:** 4416–4420.
32. Goldberg DI, Khoo JC. Stimulation of neutral cholesteryl ester hydrolysis by cAMP system in $P388D_1$ macrophages. *Biochem Biophys Acta* 1990; **1042:** 132–137.
33. Bernard DW, Rodriguez A, Rothblat GH, Glick JM. cAMP stimulates cholesterol ester clearance to high density lipoproteins in J774 macrophages. *J Biol Chem* 1991; **266:** 710–716.
34. Bailly S, Ferrua B, Fay M, Gougerot-Pocidalo MA. Differential regulation of IL-6, IL-1α, IL-1β and TNFα production in LPS-stimulated human monocytes: role of cyclic AMP. *Cytokine* 1990; **2:** 205–210.
35. Kovacs EJ. Control of IL-1 and TNF mRNA expression by inhibitors of second messenger pathways. In: Kimball EW, ed. *Cytokines and Inflammation.* Boca Raton, FL: CRC Press 1991; 87–105.
36. Brandwein SR. Regulation of interleukin-1 production by mouse peritoneal macrophages: effects of arachidonic acid, cyclic nucleotides and interferons. *J Biol Chem* 1986; **261:** 8624–8632.
37. Tannenbaum CA, Hamilton TA. Lipopolysaccharide-induced gene expression in murine peritoneal macrophages is selectively suppressed by agents that elevate intracellular cAMP. *J Immunol* 1989; **142:** 1247–1280.
38. Scales WE, Chensue SW, Otterness I, Kunkel SL. Regulation of monokine gene expression: prostaglandin $E_2$ suppresses tumor necrosis factor but not interleukin-1α or β-mRNA and cell-associated bioactivity. *J Leukocyte Biol* 1989; **45:** 416–421.
39. Kassis S, Lee JC, Hanna N. Effects of prostaglandins and cAMP levels on monocyte IL-1 production. *Agents Actions* 1989; **27:** 274–276.
40. Sung SJ, Walters JA. Increased cyclic AMP levels enhance IL-1α and IL-1β mRNA expression and protein production in human myelomonocytic cell lines and monocytes. *J Clin Invest* 1991; **88:** 1915–1923.

ACKNOWLEDGEMENT. This work supported by ONR #N00014-89-J-1130 and the RGK Foundation.

**Received 28 April 1995; accepted 8 May 1995**
